# Over-the-Counter Medicine-Seeking Behavior in Patients With Dermatophyte Infections Across Various Socioeconomic Strata: A Cross-Sectional Study

**DOI:** 10.7759/cureus.51686

**Published:** 2024-01-05

**Authors:** Nishant Choudhary, Divya Panday, Dhruv Mishra, Koushik Lahiri, Amrita Sil, Ramit Chaddha

**Affiliations:** 1 Dermatology, Chirayu Medical College and Hospital, Bhopal, IND; 2 Dentistry, LN Medical College and Research Center, Bhopal, IND; 3 Dermatology, Apollo Hospitals, Kolkata, IND; 4 Pharmacology, Rampurhat Government Medical College and Hospital, Birbhum, IND; 5 Dentistry, LNCT Medical College, Indore, IND

**Keywords:** socioeconomic class, self medication, steroid use, dermatophytosis, fungal infection, over-the-counter medication

## Abstract

Background and objective: In dermatology outpatient departments in India, dermatophytosis is the most commonly encountered dermatosis. The objective was to collect data regarding the prevalence of over-the-counter (OTC) medication, knowledge of the illness, and hygiene habits among people with dermatophyte infections across various socioeconomic classes.

Methods: At a tertiary care facility in central India, a cross-sectional study was carried out over six months. Data about socioeconomic class, hygiene routines, prior history of any type of treatment, understanding of the condition, and family history of similar illnesses were noted. A total of 551 patients were included in the study. The correlation was evaluated using Spearman's correlation coefficient (rho).

Results: Socioeconomic class had little impact on seeking dermatologist advice. Steroids were prescribed to approximately 81.8% of all patients. There was a positive correlation (rho = 0.237) between the use of steroids and the severity of the condition. Around 34% of patients took prescription medications, as recommended by a pharmacist. The use of steroids positively correlated (rho = 0.426, p<0.001) with prescriptions by pharmacists. Over-the-counter drug availability and individuals believing pharmacists were qualified to identify and treat illnesses were significant contributing causes.

Conclusions: People from all socioeconomic strata need to be made more aware of the risks associated with the inappropriate use of medications and skin problems in general. Strict regulations to control prescription drug sales and deter practitioners of alternative medicine from prescribing allopathic medications may be beneficial.

## Introduction

Dermatophytosis is the most commonly encountered dermatosis in dermatology outpatient departments in India. The prevalence rate of superficial mycotic infection is 20% to 25% globally [1]. Dermatophytosis is caused by pathogenic dermatophyte fungi. Dermatophytes are classified into six genera, namely *Trichophyton*, *Microsporum*, *Epidermophyton*, *Nannizzia*,* Lophophyton*, and *Arthroderma* [2]. The chronicity and recurrence of dermatophytosis even after proper treatment have led to mental, social, and financial disturbances among patients and a feeling of incompetence among doctors [3].

Dermatophytosis is more common in regions with a hot and humid climate [4]. The spread of infection depends on host immunity, drug penetration, drug resistance, patient compliance, and persistence of infection foci due to the sharing of fomites, irregular hygiene practices, and poor cleanliness [5-8]. The use of steroids, especially as an over-the-counter (OTC) drug, has been an important cause of poor responses to anti-fungal therapy [9,10]. Inappropriate regimens and suboptimal dosages of oral and topical antifungals prescribed by unauthorized practitioners and various other medical specialties are yet another important cause of the persistence of dermatophytosis. The cost of medication is also a factor in non-adherence by patients.

Although many epidemiological and microbiological studies have been conducted throughout the Indian subcontinent, they do not focus on the behavior and awareness of the patient [11-13]. We conducted this study to determine the prevalence of OTC medication, awareness about the disease, and hygiene habits among people with dermatophyte infections across various socioeconomic classes.

## Materials and methods

A total of 551 patients were enrolled in this cross-sectional questionnaire-based study for six months (1 July 2021 to 30 December 2021) at a tertiary care center in central India. All patients aged 12 years and older attending the dermatology outpatient department who were clinically diagnosed with dermatophyte infection and gave informed consent were included in the study. However, patients on systemic steroids for other medical conditions were excluded. Patients who were not willing to give informed consent were also excluded from the study. A potassium hydroxide (KOH) mount was made whenever deemed necessary. Permission was obtained from the Institutional Ethics Committee of LN Medical College and Research Centre, Bhopal, MP, India (approval no. 188). The study was performed per good clinical practices.

The study tool was a structured questionnaire (see Appendix A), which was constructed and validated by a dermatologist unrelated to the study. The questionnaire was handed to patients after an explanation of the purpose of the study. Any doubts regarding the questionnaire were clarified by the investigator.

Demographic data, information regarding past treatment, their understanding of the disease, family history of similar illnesses, and hygiene practices were recorded at a face-to-face interview using the structured questionnaire. A modified Kuppuswamy socioeconomic scale was used to assess socioeconomic class [14]. The Kuppuswamy scale grades socioeconomic classes by using scores based on the occupation of the head of the family, the education of the head of the family, and the total monthly income of the family [14]. The sociodemographic data recorded were age, sex, marital status, occupation, level of education, and income of the whole family.

The severity of the disease was measured in terms of involvement of the body surface area (BSA). Roughly 1% of the surface area of the body can be represented by the region of a stretched-out palm from the wrist to the tips of the fingers. Depending on the body surface area involved, it can be graded as mild (<3% BSA), moderate (3% to 10% BSA), or severe (>10% BSA).

Statistical analysis

The data was compiled and analyzed using SPSS Statistics version 21.0 (IBM Corp., Armonk, NY, USA). Data were grouped and expressed as frequency and percentage, whereas numerical data was expressed as mean ± standard deviation. The correlation between socioeconomic classes and health awareness, severity of disease, and other parameters was assessed using Spearman’s correlation coefficient (rho). A p-value of <0.05 was considered statistically significant.

## Results

A total of 551 patients were enrolled in the study. Of these, 356 (64.6%) were male, and the rest were female. Nine (1.6%) patients belonged to the upper socioeconomic group (socioeconomic class I), 246 (44.6%) belonged to the upper middle socioeconomic group (socioeconomic class II), 215 (39%) belonged to the lower middle socioeconomic group (socioeconomic class III), and 81 (14.7%) belonged to the lower socioeconomic group (socioeconomic class IV).

The socioeconomic class had no or minimal influence on seeking consultation by dermatologists (rho = 0.047; p > 0.05). Only 27 (4.9%) patients across all classes were treatment naïve, and 74 (13.4%) consulted a dermatologist before attending our OPD. In our study, 188 (34.1%) patients took medicines suggested by pharmacists. Quacks were consulted by 69 (12.5%) patients. Twenty-seven (4.9%) patients self-medicated based on medicines prescribed for skin ailments in the past or internet videos and television commercials. Self-medication was more prevalent in socioeconomic class I (Table 1). Twenty-six (4.7%) patients took medicines on the recommendations of their friends and relatives. Alternative medicine practitioners (ayurveda, homeopathy, or unani) were consulted by 16 (2.9%) patients. A large percentage of people across all socioeconomic classes consulted a pharmacist before coming to a dermatologist.

**Table 1:** Socioeconomic classes and the professional/specialist consulted Socioeconomic class I: Upper class; Socioeconomic class II: Upper middle class; Socioeconomic class III: Lower middle class; Socioeconomic class IV: Lower class

Professional/Specialist consulted	Socioeconomic class I (n=9)	Socioeconomic class II (n=246)	Socioeconomic class III (n=215)	Socioeconomic class IV (n=81)
Dermatologist	0%	21.1%	11.6%	0%
Non-dermatologist physicians (MBBS, MD/MS)	44.5%	17%	14.4%	9.9%
Pharmacist	22.2%	29.7%	31.2%	55.6%
Alternative medicine practitioners (ayurveda/homeopathy/unani)	0%	2.1%	5.2%	0%
Quacks	0%	6.5%	13.5%	29.6%
Friends or relatives	0%	7.7%	2.8%	1.2%
Self-medication	33.3%	11.8%	13.9%	2.5%
Treatment naïve	0%	4.1%	7.4%	1.2%

Out of 551 patients, 294 (53.3%) considered the disease some sort of infection, 65 (11.8%) thought their disease was an allergic reaction in response to their environment, and 18 (3.3%) considered the disease a result of their eating habits. Two patients who associated it with eating habits considered the disease to be the result of eating eggplant or oily food. Five (0.9%) patients considered it a bad omen, whereas two (0.4%) thought it was a drug reaction. And 106 (30.12%) patients admitted they didn’t know what the disease was, and one patient considered it to be a form of cancer.

The use of steroids positively correlated (rho = 0.426, p<0.001) with prescriptions by pharmacists. Steroids were prescribed by pharmacists to 93.6% of patients and by non-dermatologist doctors to 88.6%. Quacks prescribed steroids to 91.9% of patients, and 81.2% self-medicated with steroids. Steroids were prescribed most commonly through the topical route, but oral and parenteral routes were also used in some patients. The use of steroids was negatively correlated (rho = -0.176; p<0.001) with prescriptions from dermatologists. Pharmacists often suggested steroids with oral antifungals. Among the antifungals prescribed along with steroids, itraconazole was the most prescribed molecule, which was observed in 75.2% of patients. The severity of the disease increased following pharmacist prescriptions (Table 2).

**Table 2:** Correlation between severity of dermatophytosis and several parameters The correlation coefficient was evaluated using Spearman’s correlation (rho) between variables.

Parameters	Correlation coefficient (rho)	p-value	95% confidence interval	Explanation
Severity of dermatophytosis and steroid usage	0.237	<0.001	0.158 to 0.317	Significant positive correlation. The severity of dermatophytosis increases with steroid use.
Severity of dermatophytosis and pharmacist prescription	0.177	<0.001	0.096 to 0.254	Significant positive correlation. The severity of dermatophytosis increases with pharmacist prescription.
Severity of dermatophytosis and prescription by non-dermatologist	0.169	<0.001	0.0869 to 0.249	Positive correlation. The severity increases with non-dermatologist prescription.
Severity of dermatophytosis and onychomycosis	0.276	<0.001	0.197 to 0.352	Moderately high positive correlation. The severity of dermatophytosis increases with the presence of onychomycosis.
Severity of dermatophytosis and family history	0.238	<0.001	0.157 to 0.315	Moderately high positive correlation. The severity of dermatophytosis increases with the familial history of dermatophytosis.
Severity of dermatophytosis and Kuppuswamy’s socioeconomic classes	0.169	<0.001	0.0867 to 0.249	Positive correlation. The severity of dermatophytosis is higher in socio-economic classes III and IV.

About 450 patients had a prior history of treatment without consulting a dermatologist. There were various reasons behind it. One hundred and forty-two (25.8%) patients did not consider it worth spending time visiting a dermatologist and believed that it was much easier for them to get a consult from a pharmacist. Ninety participants were unable to consult a dermatologist earlier as none was available in their village or city of residence, and therefore they had to seek the opinion of whoever was available. A large number of patients (15.3%) considered pharmacists capable enough to diagnose and treat their skin condition, while 89 patients (15.8%) thought their condition wasn’t serious enough to warrant a consultation with a dermatologist. Around 8.2% of patients avoided seeking a dermatologist’s consultation as they believed the treatment suggested would be expensive to follow. The sharing of clothes and towels was seen more in socioeconomic classes III and IV (Table 3). Regular washing and ironing of clothes were noted among participants belonging to higher socioeconomic classes.

**Table 3:** Correlation between hygiene practices and socioeconomic classes Socioeconomic class I: Upper class; Socioeconomic class II: Upper middle class; Socioeconomic class III: Lower middle class; Socioeconomic class IV: Lower class

Parameters	Socioeconomic class I (n=9)	Socioeconomic class II (n=246)	Socioeconomic class III (n=215)	Socioeconomic class IV (n=81)
Percentage of patients having a daily bath	100%	95.93%	98.13%	88.75%
Percentage of patients sharing clothes	0%	32.52%	43.72%	58.02%
Percentage of patients sharing towels and soaps	66.66%	85.36%	92.09%	86.41%
Percentage of patients washing clothes regularly	100%	90.24%	76.44%	56.79%
Percentage of patients ironing clothes regularly	55.55%	14.63%	8.83%	4.93%

## Discussion

There is a lack of awareness regarding skin disorders, even among the educated masses. The present study observed no or minimal influence of socioeconomic class on seeking consultation by dermatologists (rho = 0.047; p > 0.05). Consulting a non-dermatologist doctor for their skin ailment is a common practice among the participants. Many participants understood their disease as some kind of infection, whereas few considered it an allergy or a result of their eating habits.

Approximately 22.6% of patients consulted specialists such as gynecologists, internists, and surgeons for their dermatological conditions. None of these specialists referred the patient to the dermatologist. Patients were either misdiagnosed or prescribed antifungals with steroids. This led to chronicity and an increased severity of the disease. Referral of patients for their diseases to the respective specialist at early stages can be helpful to improve the disease outcome and decrease the suffering of the patient. Misuse of all forms of steroids, especially topicals, is rampant across all socioeconomic classes in India [9]. The sale of topical corticosteroids accounts for about 82% of all sales of topical drugs in India [9]. In our study, 451 (81.8%) patients were prescribed steroids. Even though the sale of steroids without the prescription of a qualified doctor is not allowed, the OTC sale of these molecules continues to grow [10]. Strict implementation of rules that prevent the OTC sale of steroids might be helpful. Quacks were consulted by 69 (12.5%) patients. A quack is a person who practices medicine without having any formal education in modern medicine or alternative medicine and does not possess a valid medical degree or license.

Steroids modify fungal growth and morphology [15]. Following exposure to steroids, there is an upregulation of genes involved in drug resistance and biofilm production in fungi [15]. Stimulation of fungal growth has also been observed [15]. The severity of the disease was positively correlated (rho = 0.237) with the use of steroids (Table 2). Steroids should not be used to treat dermatophytosis.

The severity of dermatophytosis is worse in patients from lower socioeconomic backgrounds [6]. In a study by Ranganathan et al., 69.2% of patients were of lower socioeconomic status [6]. In the study conducted by Mahalakshmi et al., 52.6% of patients were from the lower socioeconomic class [16]. In our study, 53.7% of patients belonged to socioeconomic classes III and IV, and the severity of the disease had a positive correlation with lower socioeconomic classes (Table 2).

Regular bathing and washing of clothes help prevent fungal infections. Dermatophytosis spreads by contact with items contaminated by fungus, like towels and clothes [17]. Unhygienic practices such as sharing towels and clothes, the absence of daily baths, and not washing clothes regularly were more prevalent in patients of lower socioeconomic classes (Table 3). These can be contributing factors to the prevalence and severity of dermatophytosis among lower socioeconomic classes. While providing consultation, dermatologists should advocate hygienic practices to decrease the recurrence and severity of disease.

Three hundred and forty patients (61.7%) had a positive family history of dermatophyte infections. There was a moderately high positive correlation (rho = 0.238) between the severity of the disease and the presence of similar illnesses in the family (Table 2). This can be because of the ability of dermatophytes to spread by direct contact and through fomites [17]. All infected family members should be treated simultaneously. This would prevent the recurrence, chronicity, and severity of the disease.

Onychomycosis has been associated with the chronicity of dermatophyte infections [18]. In our study, onychomycosis is positively correlated (rho = 0.276) with the severity of the disease (Table 2). The results of this study suggest that with an increase in the socioeconomic status of people, hygienic practices tend to increase, but awareness and understanding of dermatological and medical conditions do not.

Self-medication has been observed for conditions like allergies, headaches, heartburn, and neck pain even in developed countries such as the United States of America [19]. Self-reported treatment rates range from 15% to 39% for these disorders [19]. However, in contrast to developing countries like India, developed countries such as the United States of America and the United Kingdom enforce laws that prohibit the OTC sale of medicines such as antibiotics [20].

In our study, 16 patients consulted alternative medicine practitioners. Out of these 16 patients, 15 were prescribed allopathic medicines. This practice of cross-pathy is dangerous and is on the rise [21,22]. These cross-practitioners often prescribed steroids along with oral antifungals such as fluconazole and itraconazole. The government should discourage cross-pathy and implement laws that ban such practices [21,22].

We suggest the strict implementation of laws that prohibit OTC sales of prescription drugs. Television commercials promoting medicine for common skin disorders should be discouraged [23]. Stringent laws are needed to discourage practitioners of alternative medicine from prescribing allopathic drugs. The public should be educated regarding the irrational use of drugs and their consequences. The public could be educated via social media awareness campaigns run by doctors and health organizations. Distributing educational leaflets or pamphlets to the patients can also be helpful. An increased level of understanding among patients regarding their disease is necessary to enable them to be cognizant of the severity of the disease, seek consultation with a specialized physician, and get proper treatment. This awareness regarding the harmful effects of self-medication should also be incorporated into the educational training of children right from their primary education days.

Limitations

Ours is a facility-based, cross-sectional study. The limitations of our study are that the results cannot be generalized and there could be recall bias regarding the history of past medication.

## Conclusions

People from all socioeconomic strata need to be made more aware of the risks associated with the inappropriate use of medications and skin problems in general. Strict regulations to control prescription drug sales and deter practitioners of alternative medicine from prescribing allopathic medications would be beneficial. Steroids should be avoided to treat dermatophytosis. Further studies are needed to study the various aspects of OTC medication in India.

## Figures and Tables

**Figure 1:**
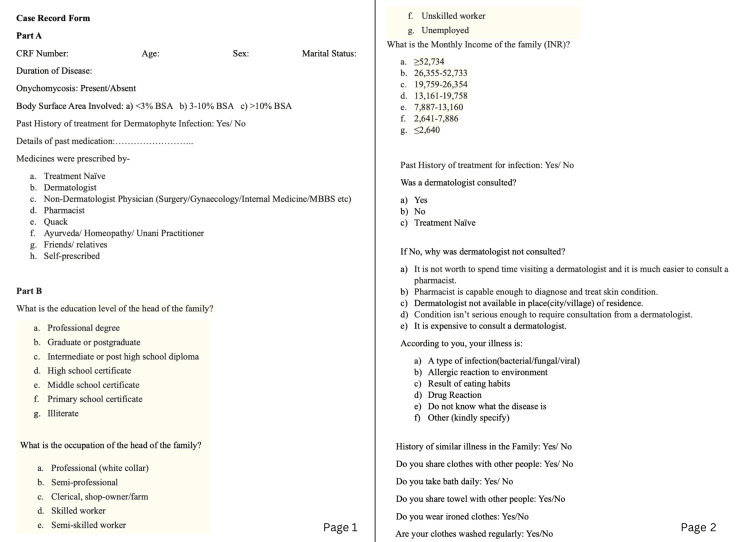
The structured questionnaire distributed to participants

## Appendices

Appendix A

Figure 1 presents the structured questionnaire that was used to interview participants and collect data for this study. This questionnaire was constructed and validated by a dermatologist unrelated to the study. It encompasses demographic data, information regarding past treatment, the patient's understanding of the disease, family history of similar illnesses, and hygiene practices. Participant responses were gathered in face-to-face interviews.
